# Double-crosslinked PNIPAM-based hydrogel dressings with adjustable adhesion and contractility

**DOI:** 10.1093/rb/rbad081

**Published:** 2023-09-09

**Authors:** Yu Cao, Longfei Wang, Xiumei Zhang, Yi Lu, Yan Wei, Ziwei Liang, Yinchun Hu, Di Huang

**Affiliations:** Department of Biomedical Engineering, Research Center for Nano-Biomaterials & Regenerative Medicine, College of Biomedical Engineering, Taiyuan University of Technology, Taiyuan 030024, China; Department of Biomedical Engineering, Research Center for Nano-Biomaterials & Regenerative Medicine, College of Biomedical Engineering, Taiyuan University of Technology, Taiyuan 030024, China; Shanxi-Zheda Institute of Advanced Materials and Chemical Engineering, Taiyuan 030032, China; Department of Biomedical Engineering, Research Center for Nano-Biomaterials & Regenerative Medicine, College of Biomedical Engineering, Taiyuan University of Technology, Taiyuan 030024, China; Department of Biomedical Engineering, Research Center for Nano-Biomaterials & Regenerative Medicine, College of Biomedical Engineering, Taiyuan University of Technology, Taiyuan 030024, China; Department of Biomedical Engineering, Research Center for Nano-Biomaterials & Regenerative Medicine, College of Biomedical Engineering, Taiyuan University of Technology, Taiyuan 030024, China; Shanxi-Zheda Institute of Advanced Materials and Chemical Engineering, Taiyuan 030032, China; Department of Biomedical Engineering, Research Center for Nano-Biomaterials & Regenerative Medicine, College of Biomedical Engineering, Taiyuan University of Technology, Taiyuan 030024, China; Shanxi-Zheda Institute of Advanced Materials and Chemical Engineering, Taiyuan 030032, China; Department of Biomedical Engineering, Research Center for Nano-Biomaterials & Regenerative Medicine, College of Biomedical Engineering, Taiyuan University of Technology, Taiyuan 030024, China; Shanxi-Zheda Institute of Advanced Materials and Chemical Engineering, Taiyuan 030032, China; Department of Biomedical Engineering, Research Center for Nano-Biomaterials & Regenerative Medicine, College of Biomedical Engineering, Taiyuan University of Technology, Taiyuan 030024, China; Shanxi-Zheda Institute of Advanced Materials and Chemical Engineering, Taiyuan 030032, China

**Keywords:** hydrogel dressings, temperature-sensitive, adjustable adhesion, adjustable contractility, scar-free healing

## Abstract

Rapid post-wound closure is necessary to avoid wound infection and promote scar-free healing when skin trauma occurs. In this study, new types of hydrogel dressings with adjustable contractility were fabricated based on *N*-isopropyl acrylamide/sodium alginate/graphene oxide (P/SA/GO). Then, the chitosan (CS) solution was used as a bridging polymer to achieve tissue adhesion to the hydrogel. The results show that the hydrogel based on poly(*N*-isopropyl acrylamide) (PNIPAM) not only has the ability to self-shrink but also can adjust the rate of shrinkage through near-infrared thermal stimulation. At the same time, high adhesion strength (7.86 ± 1.22 kPa) between the tissue and the dressing is achieved through the introduction of bridging polymers (CS), and the coating area of the bridging polymer can be adjusted to achieve regional adhesion. The mouse total skin defects experiments have shown that sutures-free wound closure in the early stages of wound healing could be obtained by adjusting the material temperature. Besides, the dressings can promote scar-free wound healing by reducing inflammatory cell infiltration and collagen deposition. These results indicate that double-crosslinked PNIPAM-based hydrogel dressings with adjustable adhesion and contractility proposed in this study provide a candidate material for achieving trackless wound healing.

## Graphical Abstract

**Figure:**
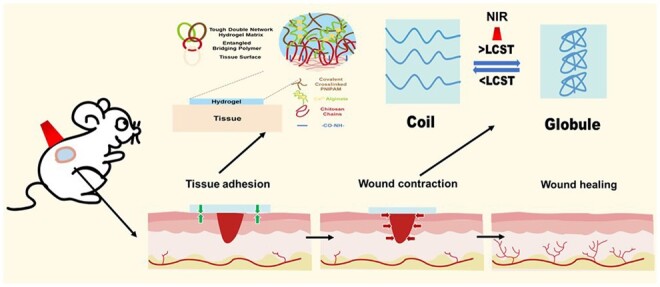

## Introduction

As the largest organ of the human body, skin is exposed to the environment and susceptible to injury in daily life, resulting in wounds [1, 2]. Although minor wounds have the ability to undergo self-healing, significant skin injuries, especially chronic wounds, can result in wound infection, tissue proliferation, and necrosis if prompt treatment is not administered [3, 4]. Wound dressings are commonly applied to the surface of wounds in order to promote healing [5–7]. These wound dressings, including gauzes [8], sponges [9–12], hydrogel dressings [13–17] and similar forms, are designed to manage bleeding and promote wound healing, as well as protect against secondary damage. They also maintain moisture in the wound area to promote the removal of excess secretion and prevent infection [13, 18]. With these treatments, common wounds can be repaired, but scars may be left, which can lead to psychological distress and social difficulties for patients [19, 20]. Therefore scar-free wound repair is necessary. Martin *et al*. have studied early wound healing in mammalian embryos and found that the actin cables form in cells at the leading edge of the wound [19, 21]. These actin cables actively contract and apply force to the wound, pulling the wound edges together. Subsequently, Allen *et al.* found that the effects of the mechanical forces could promote cell proliferation and differentiation, resulting in promoting wound recovery [22, 23]. Motivated by these studies, great progress has been made in mechanical active wound dressings [24–28]. However, most of the current mechanical active dressings achieve self-shrinkage only under specific conditions, and the shrinkage rate of the dressings cannot be adjusted [29]. Besides, existing mechanically active dressings have a certain adhesion strength, but this is the inherent property of hydrogels and is double-sided adhesion, which is inconvenient to use [30, 31]. That means, the dressings are not only easy to attach foreign bodies but easy to attach to the wound tightly, resulting in a secondary rupture of the wound in the dressing removal process. Finally, the flexibility of most mechanical active dressings is not very high, which may bring the patients a foreign body sensation. Therefore, there is an urgent requirement to prepare a flexible mechanical active hydrogel dressing with adjustable shrinkage and adhesion strength, which can finally lead to scar-free wound repair.

Poly(*N*-isopropyl acrylamide) (PNIPAM) hydrogels are the most widely studied temperature-dependent polymers [27, 32], which are widely used in contractile wound dressings [24, 26, 28, 33], drug-controlled release [34, 35] and tissue-engineered stents [36, 37]. The PNIPAM hydrogel is composed of a hydrophilic amide (-CONH-) group and a hydrophobic isopropyl (-(CH_3_)_2_CH-) side chain [38]. The low critical transition temperature (LCST) of PNIPAM hydrogel is usually 32°C [39]. At temperatures higher than LCST, the hydrophobic interactions of the PNIPAM hydrogel are enhanced, while the hydrogen bonds of the hydrophilic groups are simultaneously weakened. As a result, the PNIPAM molecules clump together, causing a conformational transition from the coil to the sphere [40]. The conformational transition causes most of the water inside the hydrogel to be discharged and the hydrogel volume to shrink significantly [41]. Since the LCST of PNIPAM hydrogel is lower than that of human skin tissue (37°C), this means that the PNIPAM wound dressing can contract under the body temperature without any treatments. Furthermore, the PNIPAM hydrogel can rapidly contract by measures of auxiliary heating, e.g. the near-infrared (NIR) [33]. The adjustable contractile mechanical active dressings are adaptive and can be used for scar-free wound repair, however, few studies have been reported on these smart dressings so far.

Sodium alginate (SA), a natural polysaccharide extracted from seaweed, is a linear copolymer formed by the β-1,4-glucoside bond of β-d-mannuronic acid (M unit) and α-l-glucuronic acid (G unit) [42–44]. Currently, there are many types of SA hydrogel wound dressings [45–47] on the market, which have the characteristics of hygroscopic, oxygen-permeable, biocompatible, high toughness and flexibility. This led us to want to add SA to PNIPAM hydrogel to improve its mechanical properties and biocompatibility. Current cross-linking of SA hydrogels is usually done by soaking CaCl_2_ to cross-link SA and then terminated by using the sodium citrate. In this way, the crosslinking speed is slow and the crosslinking is insufficient, which may enhance the time cost and material cost [48, 49]. The existing one-step crosslinking method of SA can greatly improve the defects of the two-step method. The slightly soluble calcium sulfate is directly dispersed into the gel solution, then the slow and sufficient cross-linking of SA is achieved by exploiting the slightly soluble nature of the calcium sulfate [50]. This motivates us to speculate that bringing the calcium sulfate cross-linked SA hydrogel into the PNIPAM hydrogels can not only improve the biocompatibility and mechanical properties of the hydrogel but give the hydrogels better toughness.

Graphene oxide (GO) has excellent electrical conductivity and mechanical strength. Meanwhile, the surface contains a large number of hydrophilic oxygen-containing functional groups, such as hydroxyl (–OH), carboxyl (–COOH) and carbonyl (–CO), which is beneficial for chemical functionalization modifications to enhance biocompatibility [51–53]. Most importantly, it has efficient photothermal conversion and low cost [54–57]. Therefore, the NIR effects of hydrogels can be achieved by uniformly mixing the GO components into the PNIPAM hydrogel systems. In this way, the modified hydrogel can be adjusted by the hydrogel temperature through NIR, which is helpful to realize the regulated contraction of the hydrogel.

Chitosan (CS) is selected as the bridge polymer between the modified hydrogel and the tissue to achieve the adjustable adhesion of the tissue region. Because CS is a biopolymer with a positive charge, which is easy to bond with negatively charged tissues [24, 50, 58]. The amino group on chitosan is easily combined with the carboxyl group activated by 1-(3-dimethylaminopropyl)-3-ethylcarbodiimide hydrochloride (EDC) and *N*-hydroxysuccinimide (NHS) to form an amide bond. At the same time, CS can realize physical noncovalent interactions mediated by bridging polymers, forming extensive CS networks between the gel and the tissue. Since the pKa of CS is ∼6.5, tissues (pH > 6.5) can induce gelation at deprotonation of CS chains, resulting in internal interpenetrating networks [59]. This motivates us to apply bridging polymers to the tissue surface to make the hydrogel adhere. The carboxyl group activated by CS initially reacts with hydrogels and amino groups on tissues and immediately adheres to them. At the same time, the topological adhesion of CS chain permeation is used to obtain high strength long-term stable adhesion. The advantages of this adhesive hydrogel is that the hydrogel does not attach directly to the wound and does not easily tear the wound when it is later removed, causing pain to the patient. At the same time, its adhesion is one-sided and no inconvenience will be caused after the surger.

**Figure 1.**
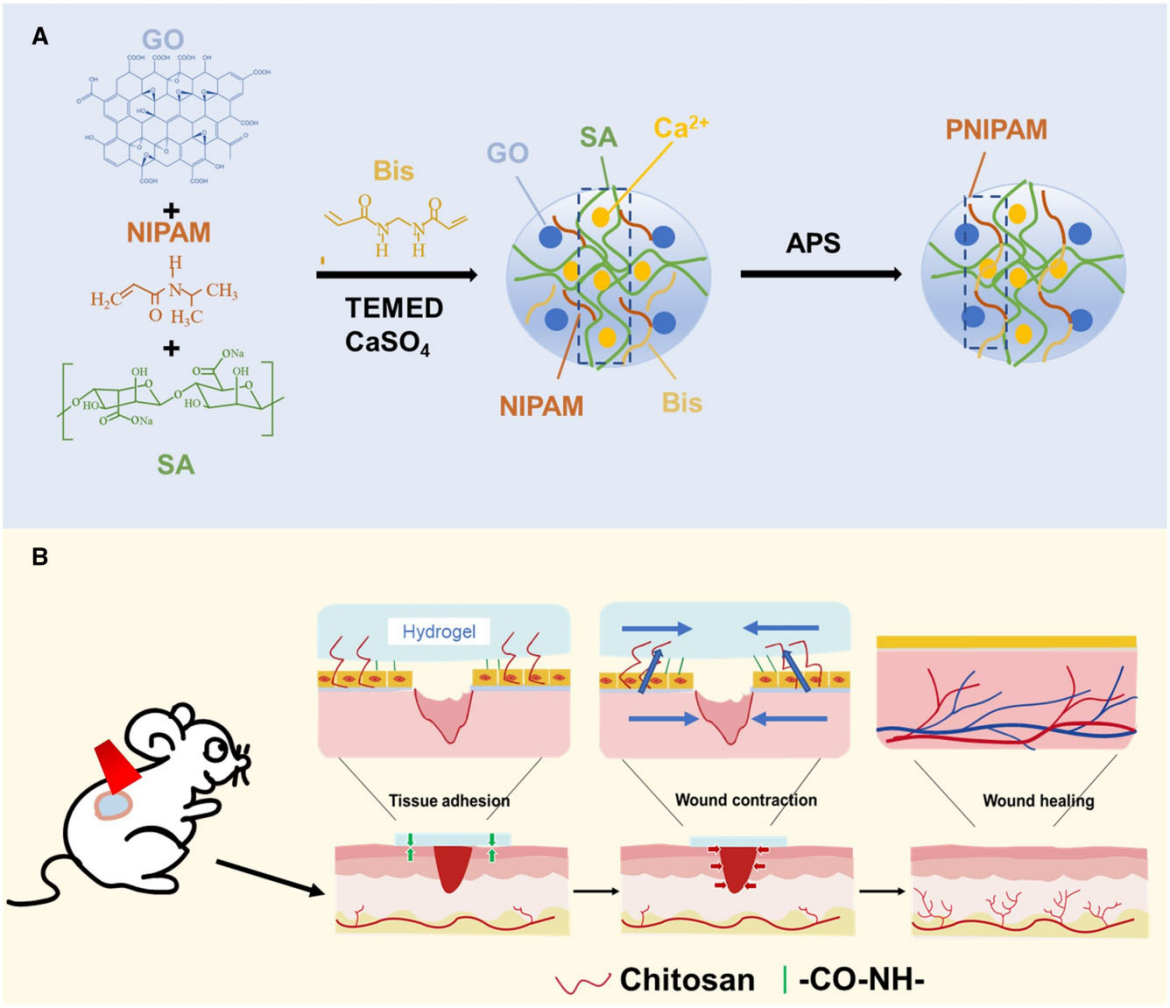
(**A**) P/SA/GO hydrogel synthesis mechanism diagram. (**B**) P/SA/GO scarless recovery mechanism.

In this study, a flexible mechanical active hydrogel dressing with adjustable adhesion and contractility is developed by composing NIPAM, SA, GO and bridging polymer CS solution together (Figure 1). To get such a hydrogel, a GO solution treated with ultrasound was first used as the solvent and it helps the hydrogels obtain excellent NIR properties. The cross-linking agent N,N’-methylene Bis (acrylamide) (Bis) was then used for cross-linking through free radical polymerization, and NIPAM was formed as the first network system so that the hydrogel has the ability to shrink automatically under heating. At the same time, slightly soluble calcium sulfate was added to the system to slow down the one-step cross-linking of SA, forming a second network system for the hydrogel. This step was designed to enhance the mechanical properties and biocompatibility of hydrogel. The prepared hydrogel can achieve relatively adjusted adhesion behavior to the skin by bridging the polymer. To validate these, the thermal response and thermal stimulation shrinkage based on the PNIPAM hydrogels were first analyzed. Subsequently, the NIR properties of hydrogels and the adjustable thermal shrinkage induced by NIR excitation were analyzed. Then, the adhesion properties of the hydrogels were confirmed by tissue adhesion experiments, and the relatively controlled adhesion properties of the hydrogels were characterized. Next, the flexibility and toughness of the hydrogels were characterized by experiments related to mechanical properties, and the biocompatibility of PNIPAM-based hydrogels was confirmed by CCK-8, live/dead cell staining and hemolysis. Finally, a mouse model of total skin defect was developed to analyze the scar-free recovery potential of flexible mechanically active hydrogel dressings through 10-day wound healing trials.

## Materials and methods

### Materials

*N*-isopropyl acrylamide (NIPAM) was purchased from Macklin Reagent Co., Ltd. (Shanghai, China). SA was purchased from Tianjin Guangfu Fine chemical Research Institute (Tianjin, China). GO was purchased from Qitaihe Baotailong Graphene new material Co., LTD (Heilongjiang). Ammonium persulfate (APS), N, N’-methylene Bis (acrylamide) (Bis) and Calcium sulfate (CaSO_4_) were purchased from Aladdin Reagent Co., Ltd (Shanghai, China). Chitosan (CS) and MES, Free acid, monohydrate (MES) were purchased from Damas-beta Reagent Co., Ltd (Shanghai, China). N,N,N’,N’-Tetramethylethylenediamine (TEMED) was purchased from Taitan Reagent Co., Ltd (Shanghai, China). Sodium hydroxide (NaOH) was purchased from Tianjin Kaitong Chemical reagent Co., LTD (Tianjin, China). EDC and NHS were purchased from RHAWN Co., Ltd (Shanghai, China). 3M was purchased from Zhidian medical equipment (China). All employed reagents were used as received. Deionized water was purified using an Ultrapure Water Machine (UPR-II, China).

### Material preparation

#### Preparation of PNIPAM hydrogel

First, 2.4 g NIAPM was weighed and added to 15 ml of deionized water, which was stirred to dissolve in an ice bath. Then add the crosslinker Bis aqueous solution 1 ml and the accelerator TEMEND 20 μl and continue stirring to mix evenly. Add another 3 ml of deionized water and finally add the initiator APS. After mixing well, the hydrogel solution was transferred to the prepared mold and placed in the refrigerator at 4°C for 24 h to form the cross-linking. Finally, the prepared hydrogel is removed from the mold and placed in deionized water for a period of time to remove the non-reactive reagents. The prepared hydrogel is called P.

#### Preparation of P/SA hydrogel

First, weigh 0.6 g SA and add it into 15 ml deionized water, put it in a 70°C water bath, and stir to dissolve. The dissolved SA solution is then cooled and transferred to an ice bath environment for stirring. Weigh 2.4 g NIAPM and add it to dissolve. Then add 1 ml Bis water solution and TEMED 20 μl and stir well. Then slowly add a 3-ml calcium sulfate solution, and finally add initiator APS. After mixing well, the hydrogel solution was transferred to the prepared mold and placed in the refrigerator at 4°C for 24 h to form the cross-linking. Finally, the prepared hydrogel is removed from the mold and placed in deionized water for a period of time to remove the non-reactive reagents. The prepared hydrogel is called P/SA.

#### Preparation of P/SA/GO hydrogel

Forty milligrams GO graphene were first passed through an ultrasonic signal generator (Shanghai BILANG INSTRUMENT Manufacturing Co. LTD) to disperse it evenly in 40 ml of deionized water. Then, 15 ml of GO aqueous solution was absorbed as a solvent to prepare the hydrogel. Subsequent preparation steps are consistent with P/SA, so there is no need to repeat it. The prepared hydrogel is called P/SA/GO.

#### Preparation of chitosan solution

Dissolve 0.214 g of chitosan in a 10-ml MES configuration buffer solution. When fully dissolved, add sodium hydroxide and adjust pH to ∼6. Then the 12 mg/ml EDC and 12 mg/ml NHS were then weighed and added to the solution in turn, stirring them to dissolve.

### Material characterization and testing

#### Surface chemistry characterization and morphology analysis

Fourier transform infrared spectroscopy (FTIR, Bruker Alpha, Germany) was used to detect the formation and changes of different chemical bonds after cross-linking hydrogels.

Before imaging, the prepared hydrogels (P, p-SA and P-SA-Go) were dried for 24 h using a freeze-dryer (CHA-S, Changzhou Guohua Electric Appliance Co., Ltd, China). The hydrogel is then extracted with liquid nitrogen, and the hydrogel section is sprayed with platinum to improve electrical conductivity. After that, the substrate morphology of the samples was observed by scanning electron microscope (SEM, JSM-7100F, JEOL Ltd, Japan) at an accelerated voltage of 3 kV.

#### Swelling ratio, porosity and thermosensitive shrinkage

The swelling ratio of the hydrogels was measured. The weight of a cylindrical hydrogel with *d* = 8 mm after freeze-out is denoted as m_1_. After that, the dried samples were soaked in PBS (pH = 7.4) at 37°C. At various times, the samples were removed and weighed, and labeled as *m*_2_. The swelling ratio is calculated by formula (1). The swelling rate at 25°C is the same as that at 37°C. At room temperature, the weight *m*_3_ and volume *v* of the hydrogel were determined, and the weight of the sample after freeze-drying was recorded as *m*_4_. The porosity is calculated by formula (2).


(1) $$\mathrm{SR}=\frac{m_{2}-m_{1}}{m_{1}}\times 100\%$$

(2) $$P=\frac{m_{3}-m_{4}}{v}\times 100\%$$

To directly observe the heat-stimulated contraction of the PNIPAM-based hydrogel, the hydrogel was placed in distilled water at different temperatures (25, 29, 33 and 37°C) and photographs of the sample were recorded using the camera. Then, to study the automatic shrinkage of hydrogel over time, the sample area change at 37°C was calculated. At the same time, the area of the sample was measured at different temperatures, and the change in hydrogel shrinkage caused by thermal excitation shrinkage was calculated. Finally, to analyze the repeatability of heat-stimulated contraction, the hydrogel was immobilized in deionized water at 37°C for 2 h, and then taken out for photos. Then it was transferred to deionized water at 25°C for 2 h and photographed again. In this way, the area change of the hydrogel is analyzed through three cycles. (The reduction in area was calculated and normalized using the Image J software.)

#### NIR properties and NIR thermal shrinkage

First, a circular hydrogel sample with a radius of 5 mm was prepared different hydrogels are irradiated with a 980-nm laser. The temperature change of the sample was recorded by an infrared thermal imager (BWT, Beijing, China). Then, P/SA/GO hydrogel was irradiated with different powers to record the temperature change. The P/SA/GO hydrogel was then exposed to a 980-nm laser for 1 min, after which the laser was turned off for natural cooling for 1 min. Three cycles were used to test the photothermal stability of the sample.

Circular and strip hydrogel samples were prepared to test the NIR temperature sensitivity of the hydrogel. First, the circular hydrogel was irradiated with 980 nm 1 W for 20 min and the temperature change was recorded with an infrared thermal imager and the area change of the hydrogel was recorded with a photographic camera. Second, the striped hydrogel sample was irradiated with a 980-nm laser at 1.5 W and the temperature change was recorded with an infrared thermal imager, followed by a photographic measurement of the striped sample width change.

#### Adhesion and mechanical properties

The instantaneous adhesion and 1 h adhesion between hydrogel and pig skin were measured by a universal testing machine (50 N, Reger RGM 6030, China). The 10 × 20 × 1.5 mm^3^ hydrogel was sandwiched between two pieces of 30 × 60 × 1.5 mm^3^ pig skin by the loading shear method. Then the instantaneous and 1 h adhesion curves were recorded.

The mechanical properties of hydrogels were evaluated by compression and tensile tests using mechanical machines (INSTRON 5848). In the stretch test, the hydrogel sample was cut into 40 × 15 × 1.5 mm^3^ strips. The single stretch rate was set to 10 mm/min. The cyclic tensile rate was set to 30 mm/min, the tensile strain range was set to 0–150%, and the cyclic count was set to 10. In the compression test, the hydrogel sample was shaped into a cylinder with a 13-mm diameter and 10-mm height. The compression strain ranges from 0% to 60% with a single compression rate of 10 mm/min. The cyclic compression rate is set to 10 mm/min, the compression strain range is 0–60%, and the period is 10 times. The tensile modulus is obtained from a linear fit of the tensile stress–strain curve in a strain interval of 10–30%. The compressive elastic modulus is derived from a linear fit of the compressive stress–strain curve over a strain range of 5–15%.

#### Biocompatibility–blood compatibility and cell compatibility

P, P/SA and P/SA/GO (diameter 8 mm, thickness 1.5 mm) were treated with normal saline at 37°C for some time. Then the material was added into 4 ml normal saline and 200 μl purchased fresh anticoagulant blood and incubated at 37°C for 1 h. Blood diluted with normal saline (200 μl) was used as the negative control, and blood diluted with distilled water (200 μl) was used as the positive control. After incubation, the material was centrifuged at 2000 rpm for 5 min and the supernatant was obtained. The absorbance at 545 nm was recorded by an ultraviolet photometer (SP-756PC, Shanghai, China). The hemolysis rate was calculated according to formula (3).


(3) $$\mathrm{HR}\left(\%\right)=\frac{{\mathrm{OD}}_{\mathrm{hydrogel}}-{\mathrm{OD}}_{\mathrm{negative}}}{{\mathrm{OD}}_{\mathrm{positive}}-{\mathrm{OD}}_{\mathrm{negative}}}\times 100\%$$

where OD_hydrogel_, OD_negative_ and OD_positive_ denote the measured absorbance of the hydrogel sample, negative control and positive control, respectively.

The toxicity of P, P/SA and P/SA/GO hydrogels on L929 cells was assessed by cell-life/death fluorescence staining followed by CCK-8 testing. The effect of hydrogel on cell proliferation was characterized. First, anhydrous gel samples were used as a control group. The sterilized P, P/SA and P/SA/GO were immersed in Dulbecco’s Modified Eagle Medium. The sample was then placed in a 24-well plate overnight. L929 cells were inoculated on a hydrogel and incubated with 5% CO_2_ at 37°C for 1, 3 and 5 days. Live and dead cells were stained using a double stain with calcein-AM/PI and ethidium homodimer (Thermo Fisher, USA), and cell images were collected with a microscope (Nikon, Tis, Japan). Based on the obtained images, the cell survival rate (the proportion of live cells) was calculated using Image J software. The cytotoxicity of the hydrogel was also assessed using a cell counting kit (CCK-8) at Day 1, 3 and 5. Absorbance at 450 nm was measured using a Biorad iMark (Biorad iMark, USA).

#### *In vivo* wound healing experiments

Evaluation of PNIPAM-based hydrogels for wound healing *in vivo*. Sixteen male Kunming mice (∼6 W, 30 g, skin temperature ≈37°C) were selected for the full-layer skin defect model and randomly divided into four groups (control group, P/SA group, P/SA/GO group and P/SA/GO NIR group). In short, the operation was performed under sterile conditions. After an intraperitoneal injection of chloral hydrate (0.4 mg/kg) to anesthetize the mice, the hair on the back was removed with a hair removal cream, and a full-layer circular skin wound with a diameter of 6 mm was formed on the mice's back with a hole punch. The skin wounds of the experimental group were then covered with water gel and coated with 3M dressings, while the wound dressings alone were applied to the control group. In the P/SA/GO NIR group, the dressing was placed on the wound, the hydrogel was irradiated with a 1-W 980 nm laser, and the temperature was recorded with an infrared imager for 5 min. After the surgery, the mice were left alone in their cages with free movement and access to food. The dressings were changed on the third and fifth days. During the dressing change phase, only hydrogel was applied instead of 3M dressing, while the control group was still dressed with 3M dressing. Wound images were recorded by the camera at 0, 3, 5, 7 and 10 days, and the wound area was measured using Image J. On Day 10, the mice were killed and the tissue surrounding the wound was removed. All animal experiments were approved by the Biomedical Ethics Committee of the Taiyuan University of Technology (TYUT-202305020). Mice are kept by state regulations on animal control.

### Statistical analysis

Statistical analysis was performed to determine whether or not the differences between the groups were statistically significant. The mean standard deviation of data from independent parallel experiments was calculated. For statistical analysis, SPSS software was used. Significance was accepted at *P < 0.05, statistically higher significant at **P < 0.01, and most significant at ***P < 0.001.

## Results and discussion

### Characterization of P/SA/GO

**Figure 2.**
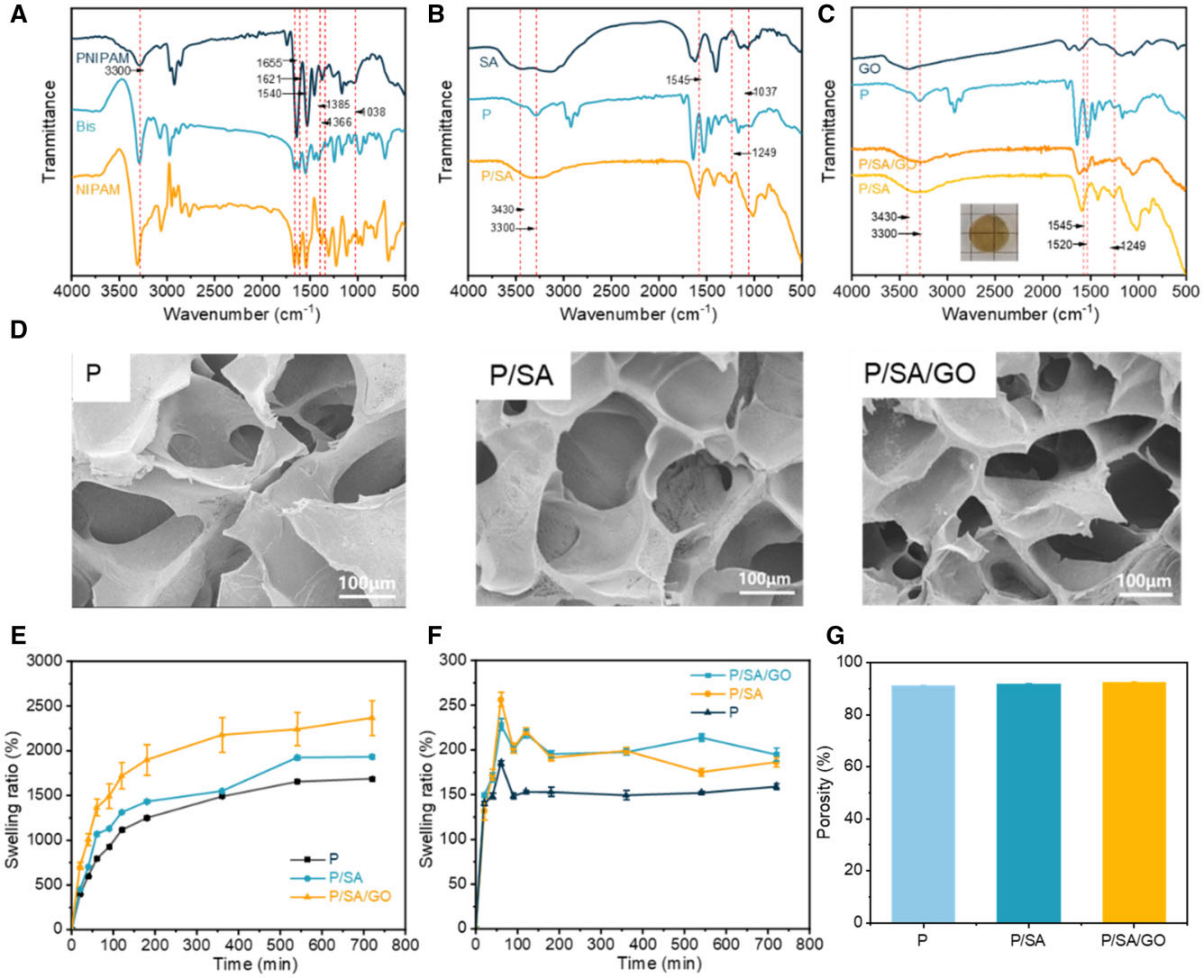
(**A**) FTIR spectra of PNIPAM hydrogel. (**B**) FTIR spectra of P/SA hydrogel. (**C**) FTIR spectra of P/SA/GO hydrogel. (**D**) SEM of PNIPAM-based hydrogels. (**E**) Swelling curve of PNIPAM-based hydrogel at 25°C. (**F**) Swelling curve of PNIPAM-based hydrogel at 37°C. (**G**) The porosity of PNIPAM-based hydrogels.

FTIR spectroscopy was used to characterize the functional groups of SA, GO, Bis, NIPAM, P, P/SA and P/SA/GO. It was found that the characteristic peaks of the isopropyl group were at 1366 and 1385/cm [60]. The stretching vibration absorption peak of C=O and the bending vibration absorption peak of N–H in amide groups of NIPAM and MBA were at 1655 and 1540/cm, respectively [61]. Compared with Bis molecules, the disappearance of the C–H bond peak of the olefin group at 3300/cm and C=C double bond peak at 1621/cm [62] in P confirmed the formation of the cross-linked network (Figure 2A). And the peaks (1037, 1249, 1545, 3301 and 3430/cm) are observable in the spectrum of P/SA (Figure 2B). The appearance of these bands strongly suggests that P/SA is successfully cross-linked. It is worth noting that the peaks belonging to C=O stretching shifted to a lower number of 1646/cm. It was reported the interpenetrating polymer network hydrogels were formed via hydrogen bonding between poly (acrylamide) and poly (acrylic acid) [63]. Considering the similar architecture and chemical composition, in the present work, the shift of the C=O stretch could be attributed to the presence of hydrogen bonding interactions between the PNIPAM and SA chains. Finally, FTIR spectra of P/SA and P/SA/GO hydrogels did not change, but GO components were evenly distributed in the hydrogels (Figure 2C).

Since the porous structure of the hydrogel helps maintain a moist environment, remove excess exudates, cool the wound surface and ensure adequate oxygen delivery, SEM was used to observe the differences in the microstructure of P, P/SA and P/SA/GO. As shown in Figure 2D, the PNIPAM-based hydrogel system exhibits a stable, homogeneous and interconnected 3D pore structure. However, with the introduction of SA and GO, the pore size of the hydrogel shrinks. It can be inferred that the pore size of PNIPAM-based hydrogels is closely related to the degree of cross-linking: as SA is introduced, P/SA interpenetration networks are formed and cross-linking becomes denser. The added GO fills the pores of the hydrogel, resulting in a denser pore structure of the P/SA/GO hydrogel. The hydrophilicity of the PNIPAM hydrogel can absorb the fluid produced at the wound site. The addition of SA improves the hydrophilicity of PNIPAM hydrogel. To verify it, the swelling rate of the hydrogel was measured. As shown in Figure 2E, the hydrogel reached swelling equilibrium at 600 min at 25°C. With the addition of SA, the swelling rate of the hydrogel increased, in agreement with the expected result. At the same time, the swelling rate of hydrogel at 37°C was much lower than that at 25°C, but similarly, the swelling rate of hydrogel added with SA was higher than that of pure PNIPAM (Figure 2F). This was attributed to the addition of SA, which improved the hydrophilicity of the composite hydrogel. In addition, the temperature sensitivity of PNIPAM hydrogel was retained by the hydrogel's swelling property.

### PNIPAM-based hydrogel shrinkage by heat stimulation

**Figure 3.**
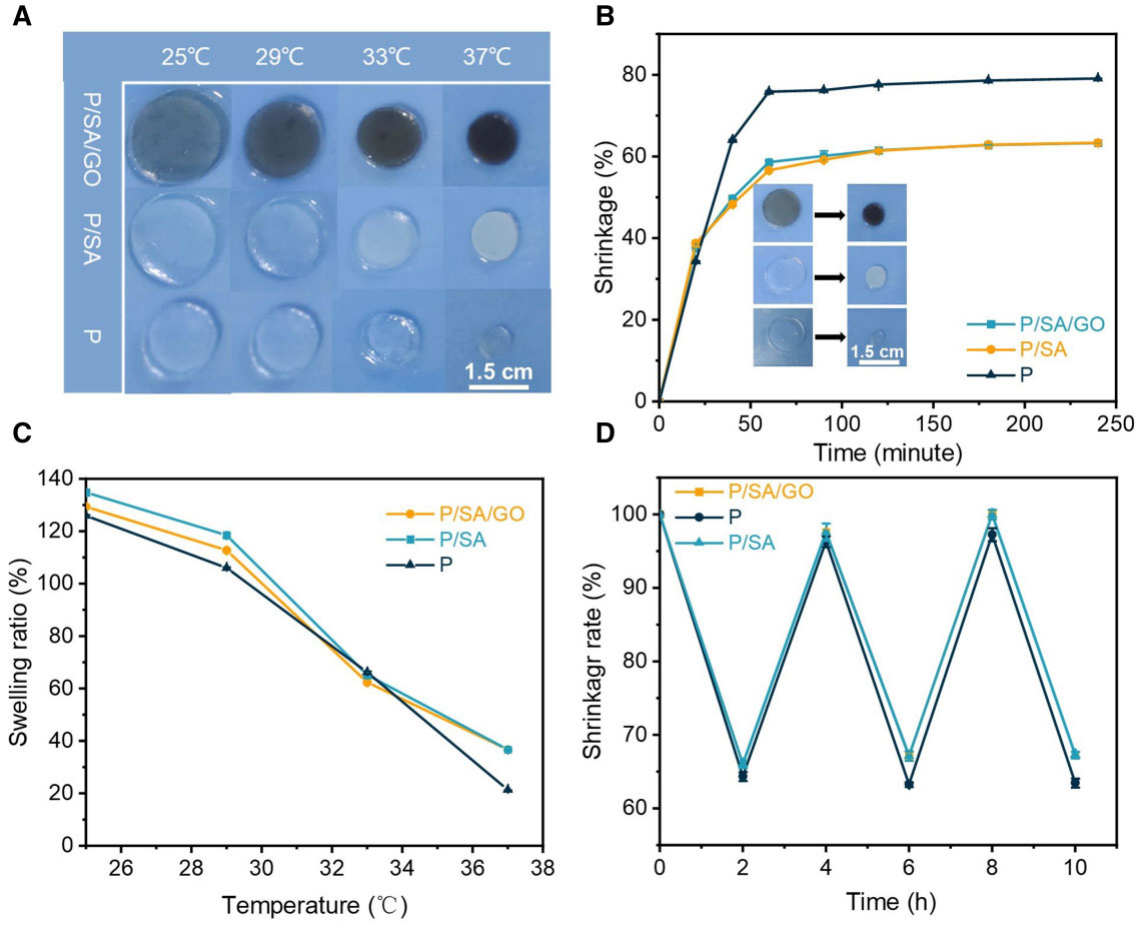
(**A**) Volume shrinkage diagram of the hydrogel at different temperatures. (**B**) Shrinkage curve of the hydrogel at 37°C. (**C**) Shrinkage size of the hydrogel at different temperatures. (**D**) Contraction and relaxation cycles of the hydrogel at 37°C and 25°C.

The LCST of PNIPAM is 32°C [39], beyond which the polymer chain can be deformed from a hydrophilic state to a hydrophobic state, and thus exhibit automatic shrinkage behavior. P, P/SA and P/SA/GO exhibit typical thermal stimulation interaction (Figure 3A). To verify the thermo-stimulated auto shrinkage of PNIPAM-based hydrogel, auto shrinkage was measured by moving the hydrogel from 25°C to 37°C (Figure 3B). Significant shrinkage of the hydrogel is observed before 40 min, reaches a maximum at 60 min, and then remains relatively stable for the rest of the time. The expansion equilibrium of PNIPAM-based hydrogel was measured at different temperatures (Figure 3C). As the temperature increases, the area of all the hydrogels shrinks. In addition, the higher the temperature, the larger the change in the area when the time is consistent, suggesting that the rate of change in the hydrogel area can be controlled by tuning the temperature. Finally, changes in hydrogel swelling balance were also observed during repeated cycles of 25–37°C (Figure 3D). These three cycles indicate the repeatability of the automatic contraction of PNIPAM-based hydrogels by thermal stimulation. This suggests that PNIPAM-based hydrogels can be used as a carrier for drug release and can be reused. However, the surface morphology of PNIPAM hydrogels was damaged, and the surface morphology of the other two hydrogels did not change significantly, indicating that the modified composite hydrogels had better morphological stability (Supplementary Figure S1).

### PNIPAM-based hydrogel contracted by NIR heat stimulation

**Figure 4.**
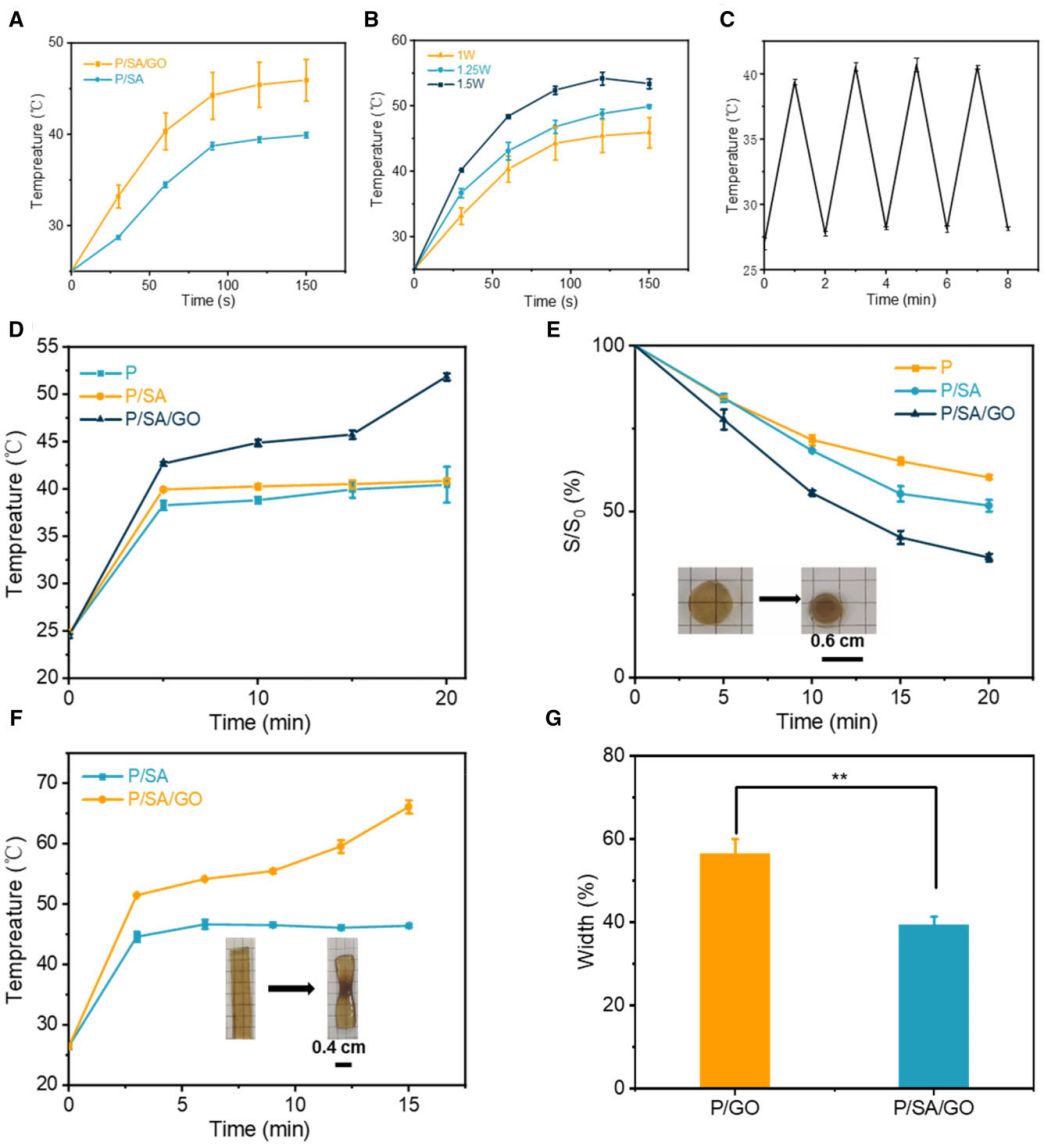
(**A**) NIR performance of the hydrogel at 1 W power. (**B**) NIR performance of P/SA/GO hydrogels at different powers. (**C**) Reciprocating cycle experiment at 1 W power. (**D**) The temperature of the circular hydrogel changes with time under NIR irradiation. (**E**) The area of the circular hydrogel changes with time. (**F**) The temperature of the strip hydrogel changes with time under NIR irradiation. (**G**) Hydrogel width change after 20 min of strip hydrogel (**P < 0.01).

As shown in Figure 4A, the temperature of the hydrogel systems increases under laser irradiation. Among them, the temperature of the hydrogel added with GO increased significantly, indicating the excellent NIR properties of P/SA/GO. Meanwhile, with the increase of power, the temperature of the PNIPAM/SA/GO hydrogel systems increased obviously, and with the increase of power, the rate of heating becomes faster. but the highest temperature does not exceed 55°C (Figure 4B). Because the PNIPAM-based hydrogel turns white after absorbing heat and removes large amounts of water, it limits temperature increases (Supplementary Figure S2). The switching cycle test demonstrates that PNIPAM/SA/GO is photothermal stable (Figure 4C). Since PNIPAM hydrogel dressings rely on the heat conduction of body temperature, the hydrogel temperature will vary slowly, which will limit its use. NIR regulation is a very appropriate means. At the same time, the NIR-induced temperature does not cause skin burns due to the PNIPAM property. Next, circular and strip hydrogel samples were prepared to validate the controlled NIR thermal stimulation contraction of the PNIPAM hydrogel. First, a circular hydrogel sample was irradiated with 1 W power. The temperature curves are shown in Figure 4D. During the experiment, the highest temperature reached 51.9°C. As shown in Figure 4E, the area of P/SA/GO shrinks to 36.16 ± 1.09% of the original area, which was significantly less than that of P/SA (51.80 ± 1.71%) and P (60.30 ± 0.53%). The temperature diagram of hydrogel and the actual change diagram in the process of NIR are shown in Supplementary Figures S3 and S4. The striped hydrogel sample was then irradiated with a power of 1.5 W. The temperature curve is shown in Figure 4F. And the width of the P/SA/GO hydrogel becomes 39.31 ± 1.99% of the original width, and the P/SA hydrogel becomes 56.44 ± 3.64% of the original width, as shown in Figure 4G. This indicates that the control of the hydrogel contraction rate can be achieved using the near-IR for the hydrogel samples prepared in this work. In addition, the characterization of samples with different shapes suggests that hydrogels have the potential to provide wound contraction for wounds with different shapes.

### Tissue adhesion of PNIPAM-based hydrogel

**Figure 5.**
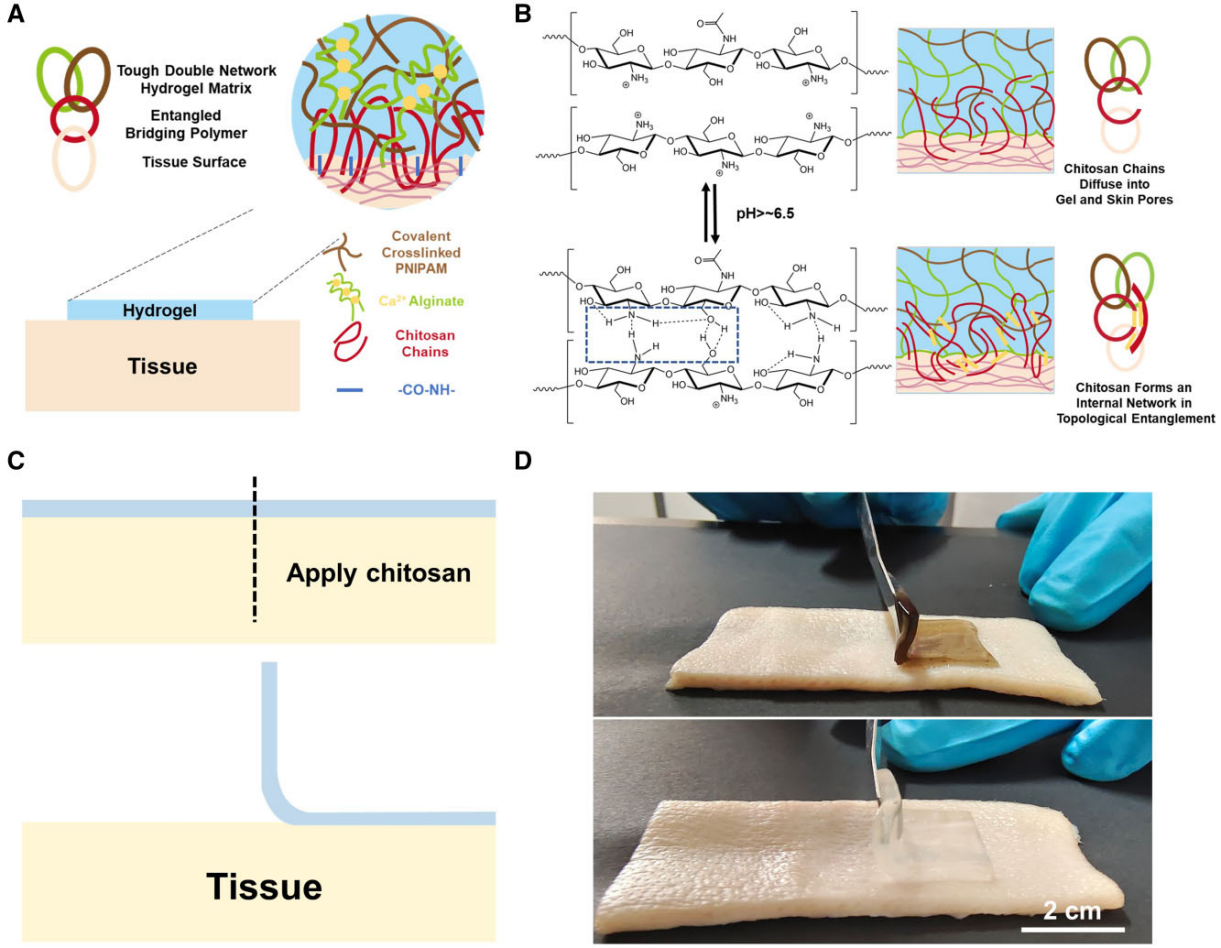
(**A**) Schematic diagram of the adhesion of the hydrogel. (**B**) Diagram of the hydrogel adhesion mechanism. (**C**) Schematic diagram of controlled adhesion. (**D**) Physical drawing of controlled adhesion.

To convert the thermally excited volume contraction of the hydrogel into a wound dressing that gives a contraction force at the wound edge, the hydrogel needs to have some adhesion to the tissue. The adhesive properties of the hydrogel prepared in this work were obtained as follows: a small amount of the prepared chitosan bridging polymer solution was applied to the pig skin. The adhesion diagram of the tough interpenetrating network prepared in this paper is shown in Figure 5A. Under the action of a coupling reagent, the amino group on the transient chitosan binds to the carboxyl group on the skin tissue and SA hydrogel to form an amide bond and achieve instantaneous adhesion. The CS network then permeates into the tissue and hydrogel, forming topological entanglements. At the same time, the tissue surface pH ≈ 6.5 was higher than the pKa of CS. The chitosan is protonated to form a gel (Figure 5B). In this way, a long-term stable adhesion effect is achieved. This mode of adhesion allows us to control the adhesion of hydrogels. Apply a little CS solution to the pig skin tissue on the right side of the hydrogel and leave the left side untouched. This allows only the right tissue and hydrogel to adhere (Figure 5C and D). This led us to speculate on applying a CS solution to the edge of the wound. Allow the hydrogel to adhere to intact, good skin tissue around the skin without direct contact with the wound. In this way, the pain of the patient will be relieved to some extent during the stripping process, without causing a tear in the wound. Moreover, this indirect means gives the adhesive properties of hydrogel in the actual application process can be one-way, which is greatly convenient for the use of patients (Supplementary Video S1).

**Figure 6.**
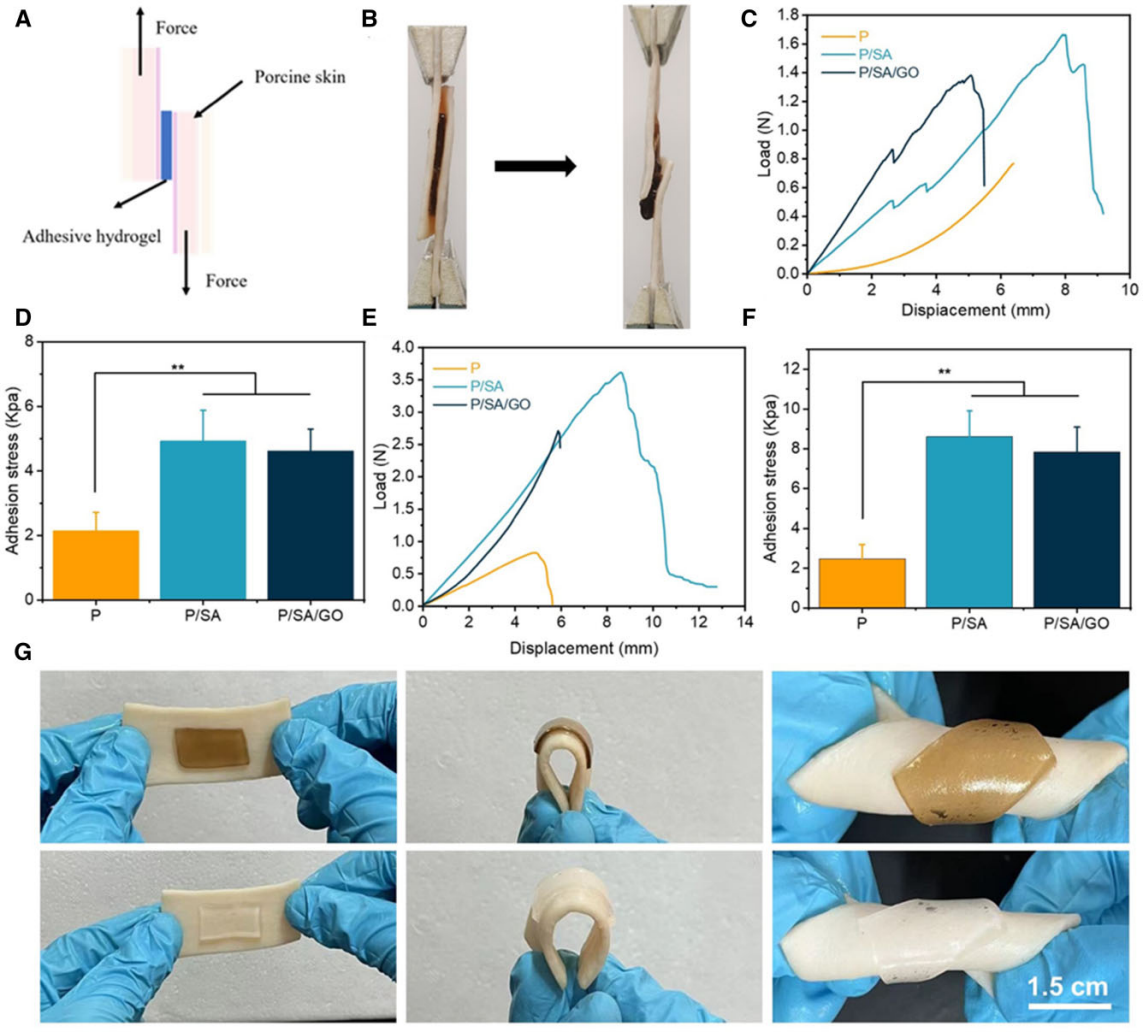
(**A**) Schematic diagram of piggy-on shear. (**B**) Schematic diagram of the material. (**C**) Instantaneous adhesion load and displacement curves. (**D**) Instantaneous adhesion stress. (**E**) One hour adhesion load and displacement curves. (**F**) One hour adhesion stress. (**G**). Adhesion, bending adhesion and torsion adhesion diagrams of hydrogel porcine skin (**P < 0.01).

At the same time, the characteristics of mechanically active hydrogel dressings require them to meet certain adhesion strength. The hydrogel was pressed against the pig skin and the instantaneous adhesion load and displacement curves of the hydrogel were measured as shown in Figure 6A. The adhesion stress of the hydrogels was then calculated at 2.14 ± 0.58, 4.94 ± 0.95, and 4.62 ± 0.68 kPa, indicating that the addition of SA improves the adhesion properties of the hydrogel (Figure 6B). It is speculated that the introduction of SA improves the toughness of the hydrogels and makes the composite hydrogels form a tough dissipative hydrogel network. At the same time, the dissipative network of SA enables the bridging polymer CS to penetrate into the interpenetrating network better. Secondly, we measured the 1 h adhesion load and displacement curves of the hydrogel (Figure 6C). Then 1 h adhesion stress of hydrogel was calculated, as shown in Figure 6D, where the adhesion stress of P, P/SA and P/SA/GO were 2.48 ± 0.71, 8.61 ± 1.3 and 7.86 ± 1.23 kPa, respectively. These results indicate that the hydrogel systems prepared in this work have rapid and excellent adhesion, and with the extension of time, the adhesion performance is stronger (Figure 6D). This is because, with the extension of time, CS penetrated the hydrogel and tissue and then gelated, forming a hydrogel interpenetrating network. Interestingly, the adhesion of P/SA and P/SA/GO hydrogels at 1 h was superior to that of P-D-C/A/W [64] (5.5 kPa underwater adhesion strength) and triple mesh gelatin/PVA composite hydrogels [65] (Gel-CG6, 7.7 kPa adhesion strength) reported in previous studies. This suggests that not only can the composite hydrogel in this paper adhere to wounds like other clinical glues, but the higher adhesion strength helps accelerate wound closure through the automatic contraction of thermal stimulation, thus promoting wound repair. Finally, the composite hydrogel will not fall off from fresh pig skin tissue, whether bent, twisted or even under water rinse, which greatly provides convenience for patients (Figure 6G, Supplementary Video S2).

### Mechanical properties of PNIPAM-based hydrogel

**Figure 7.**
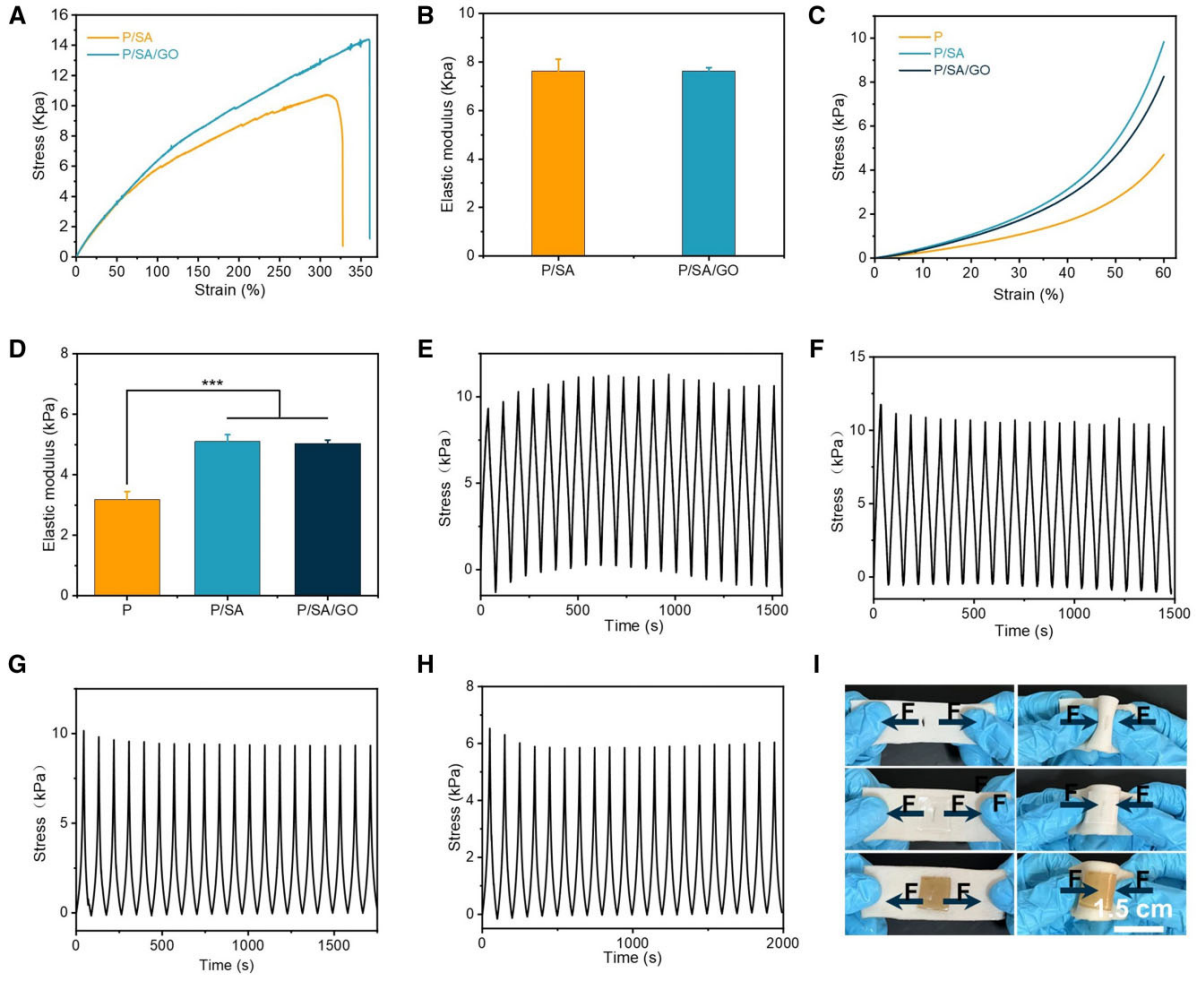
(**A**) The tensile stress–strain curve of the hydrogel. (**B**) Tensile modulus. (**C**) Compression stress–strain curves of hydrogels. (**D**) Compression modulus. (**E**) Cyclic stretching of the P/SA hydrogel. (**F**) Cyclic stretching of the P/SA/GO hydrogel. (**G**) Cyclic compression of the P/SA hydrogel. (**H**) Cyclic compression of the P/SA/GO hydrogel. (**I**) Stretching and bending images of hydrogels on porcine skin wounds (***P < 0.001).

Ideal wound dressings require appropriate adhesion strength and proper mechanical properties [66, 67]. The double-crosslinked network structure introduced by SA significantly improves the toughness and mechanical strength of the hydrogels. When the tensile modulus of the pure PNIPAM hydrogel could not be measured, the tensile modulus of the P/SA hydrogel reached 7.62 ± 0.50 kPa and that of the P/SA/GO hydrogel reached 7.62 ± 0.16 kPa (Figure 7A). At the same time, the elongation at the break of the two composite hydrogels reached more than 300% (Figure 7B). All types of hydrogels can be compressed up to 60% deformation without breaking. The compression modulus of PNIPAM-based hydrogels is significantly improved compared to pure PNIPAM hydrogels. The compression modulus of the P/SA and P/SA/GO hydrogels was 5.10 ± 0.23 and 5.01 ± 0.12 kPa, respectively, significantly higher than that of the pure PNIPAM hydrogel at 2.98 ± 0.27 kPa (Figure 7C). The PNIPAM-based hydrogels exhibit remarkable toughness and resiliency recovery and can withstand prolonged periods of compression and stretching. When the compression strain is 60% and the tensile strain is 150%, the stress loss of the hydrogel is <20% after 10 stretching or compression cycles (as shown in Figure 7D). After being compressed to 60% of its initial height, the PNIPAM-based hydrogel can quickly recover to its initial height after the compression load is removed. When the P/SA hydrogel and P/SA/GO were stretched to 150% of their initial length, the hydrogel returned to its initial state after the removal of the tensile load (Figure 7E–H). The actual diagram of cyclic stretching and cyclic compression of P/SA/GO is shown in Supplementary Figure S5. These mechanical properties (elasticity and toughness) enable PNIPAM composite hydrogels to better meet practical needs, such as bending and torsional adhesion at wound sites (Figure 6G). In addition, more importantly, the PNIPAM composite hydrogel in this study can also resist the influence of wound tension. Create a mechanical microenvironment for the wound similar to normal skin (Figure 7I). Often, an exposed skin wound is susceptible to the strain shown in Figure 7I, resulting in scarring. The P/SA and P/SA/GO hydrogels studied in this paper can create a suitable microenvironment for wounds due to their good adhesion strength and flexibility [68, 69]. And reduce scarring by reducing the effect of tension on wound healing. And reduce scarring by reducing the impact of tension on wound healing. Finally, the above mechanical properties and adhesion properties indicate that PNIPAM composite hydrogel has great potential for use in the wound of the moving part, and it can withstand the reciprocating motion of the finger joint (Supplementary Videos S3 and S4).

### Biocompatibility of PNIPAM-based hydrogels

**Figure 8.**
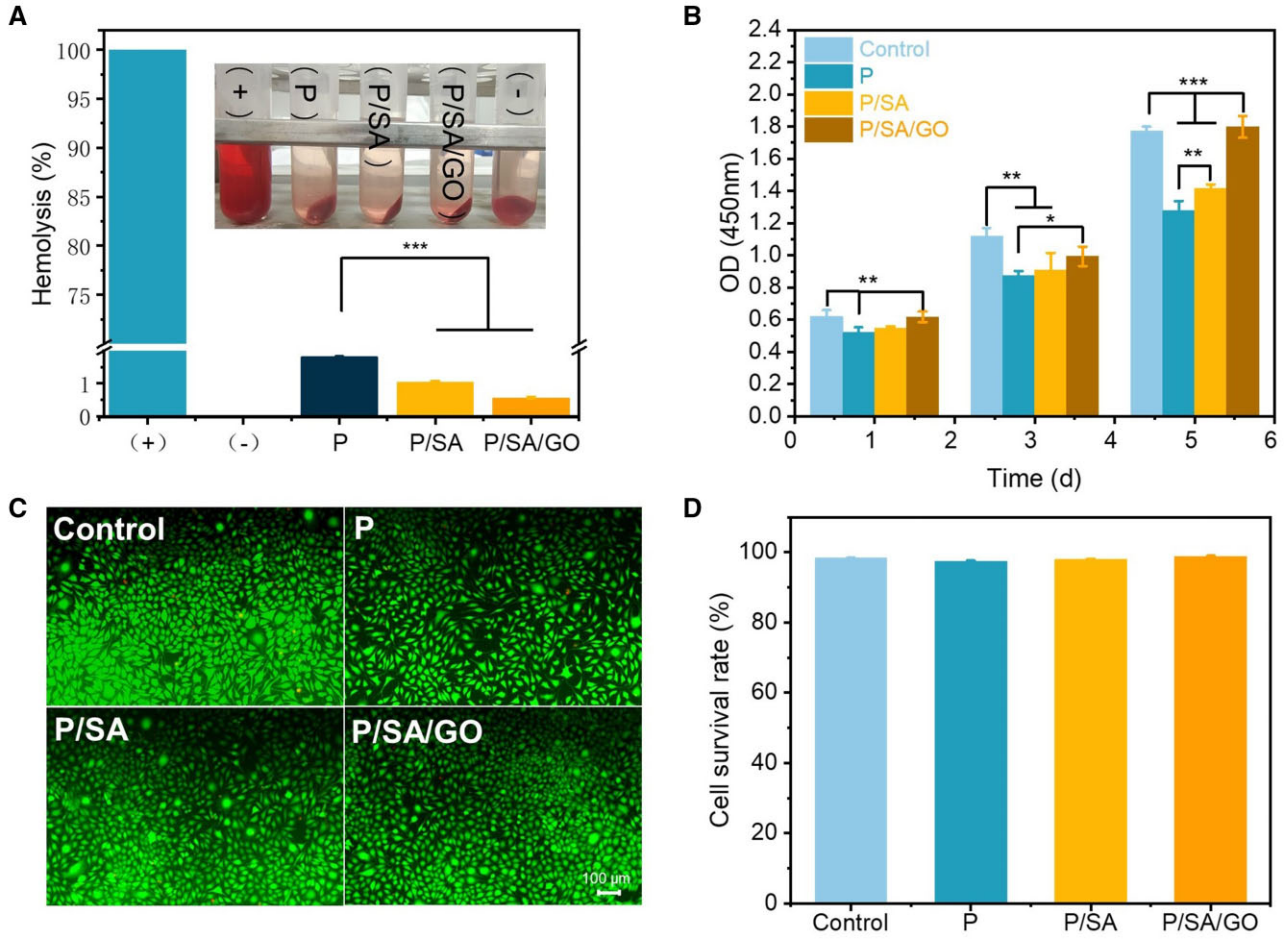
(**A**) Blood compatibility of hydrogels. (**B**) CCK-8 results at 1, 3 and 5 days. (**C**) Live/dead fluorescent staining images of fibroblasts at 5 days. (**D**) The quantitative cell survival rate from images (c) (*P < 0.05, **P < 0.01, ***P < 0.001).

The biocompatibility of the hydrogel was analyzed using blood compatibility and cell compatibility. Good biocompatibility is an important prerequisite for hydrogels to promote wound healing [31, 70]. For blood compatibility, hemolysis results showed that the hemolysis rates of PNIPAM, P/SA and P/SA/GO hydrogel were 1.81 ± 0.02%, 1.04 ± 0.02% and 0.56 ± 0.03%, respectively (Figure 8A). All values were lower than the standard hemolysis rate (4%) of the material in ISO 2-10993, indicating that PNIPAM-based hydrogel had excellent blood compatibility. For cytocompatibility, CCK-8 results showed that L929 fibroblasts in all groups showed obvious proliferation during 1–5 days, and P/SA/GO showed the best cytocompatibility in four groups (Figure 8B), confirming that the introduction of SA and GO components promoted cell proliferation. In the live/dead staining results (Figure 8C; Supplementary Figure S6), large green fluorescence spots (live cells) and a few red fluorescence spots (dead cells) were observed in all groups at 5 days. The survival rate of living and dead cells on the fifth day of quantitation showed that the cell viability of all groups reached more than 95%, with no significant difference (Figure 8D), which again confirmed the excellent biocompatibility of PNIPAM-based hydrogel.

### The traceless healing of a wound

**Figure 9.**
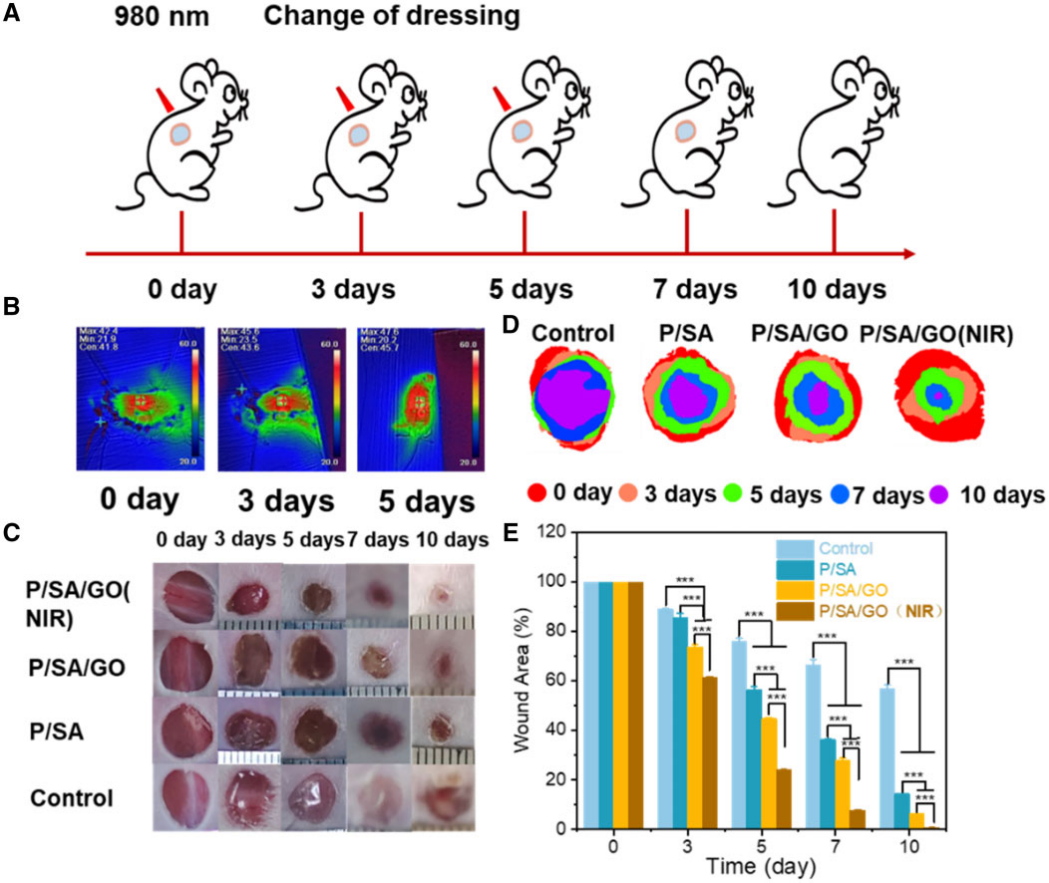
(**A**) Schematic diagram of wound management. (**B**) Images of the highest temperature of hydrogel exposed to NIR for 5 min at 0, 3 and 5 days. (**C**) Photograph of the wound. (**D**) Images of wound stacks. (**E**) Quantitative analysis of wound area (***P < 0.001).

**Figure 10.**
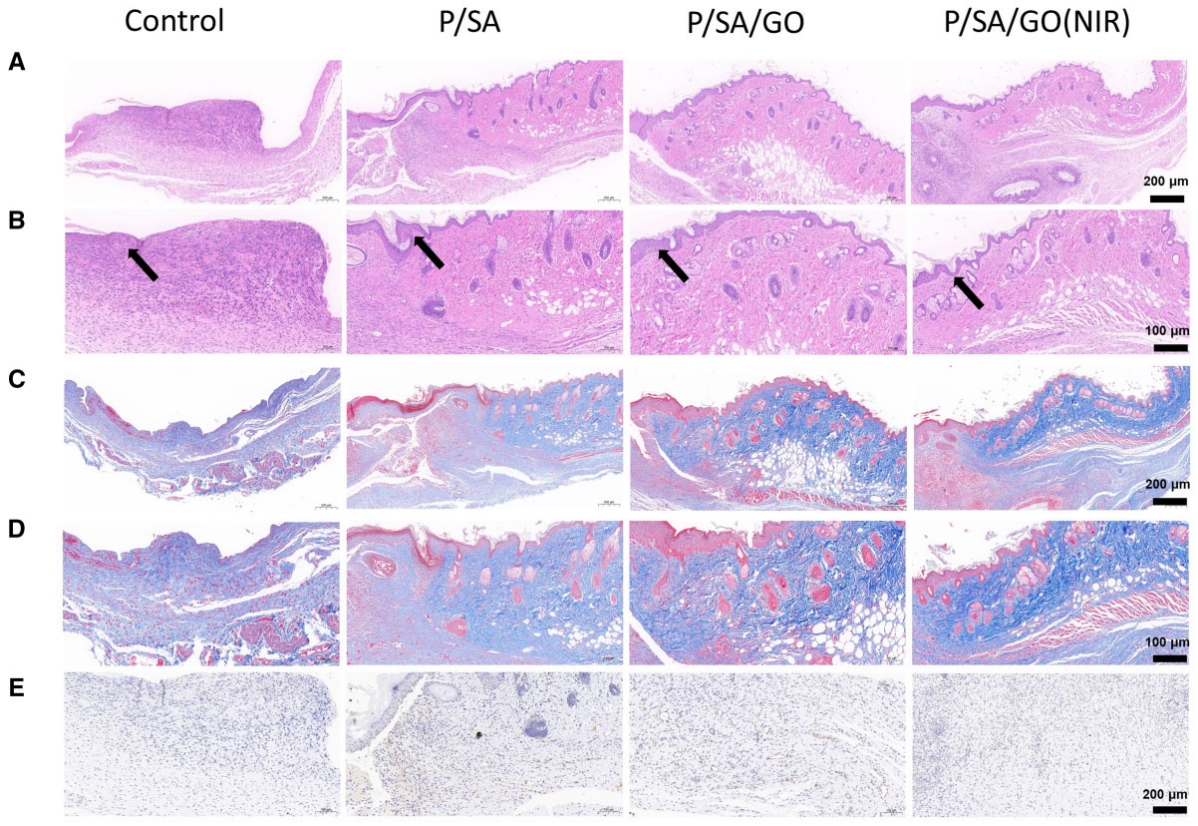
(**A**) HE staining. (**B**) Enlarged H&E staining (the black arrow indicates the tissue that forms the skin at the scar). (**C**) Masson staining. (**D**) Enlarged images with Masson staining. (**E**) Staining of vascular CD31.

To evaluate the ability of P/SA/GO to promote wound healing, a *in vivo* full-layer skin defect model was used. As shown in the figure, the wound dressing was changed in each group at 3, 5 and 7 days, respectively (Figure 9A). Among them, the P/SA/GO (NIR) group refers to the application of P/SA/GO hydrogel and the irradiation of P/SA/GO with near infrared for 5 min to stimulate the thermal trigger of the hydrogel. The highest temperature of its irradiation process is lower than 50°C as shown in Figure 9B. It seems no harm to the mice. As shown in Figure 9C, the wound area of each group gradually decreased with time. And the wound area of P-SA-GO (NIR) was the smallest, indicating that its wound healing effect was the best. The stacked placement results reconfirmed the ability of P-SA-GO (NIR) to promote wound healing (Figure 9D). Among them, the wound area of P/SA/GO (NIR) Day 3 became 61.28 ± 0.40% of the original wound area. The wound area is significantly smaller than the control group (88.98 ± 0.27%), P/SA (85.51 ± 1.79%) and P/SA/GO group (73.78 ± 0.89%). P/SA/GO (NIR) Day 7 wound area was 7.81 ± 0.19% of the original wound area, basically achieving full wound healing. The wound area of the other groups was 66.30 ± 2.31% in control group, 36.30 ± 0.25% in P/SA group and 28.18 ± 0.69% in P/SA/GO group, respectively (Figure 9C). Finally, the wounds of the P/SA/GO(NIR) group recovered basically on the 10th day, and the skin around the wounds was consistent with that of normal mice, with no scar left. This is mainly attributed to the automatic contraction of NIR induced thermal stimulation to accelerate wound closure and the synergistic action of SA to inhibit the inflammatory response and promote angiogenesis. To observe the formation of granulation tissue, H&E staining was performed, as shown in Figure 10A. The results showed that the hydrogel group had obvious granulation tissue formation, while the control group had less, and the hydrogel group had a small amount of inflammation. In contrast, the control group showed a wide range of significant inflammation, indicating that the hydrogel can effectively reduce the wound inflammatory response (Figure 10B) [71]. At the same time, it can be seen from the HE staining diagram that the epithelial tissues formed in the P/SA/GO(NIR) group were the thinnest and similar to normal skin tissues. The other two groups formed thicker tissue than normal, while the control group had not even formed epithelial tissue. Abnormal thickening of the formed epithelial tissue compared to normally indicates scar formation, while P/SA/GO(NIR) produces skin tissue that is about the same thickness as normal skin. This shows that P/SA/GO(NIR) group has basically achieved traceless repair. Excessive production and deposition of collagen increase scar volume and leads to the formation of keloids [72]. Therefore, collagen deposition at the wound site was further assessed by Masson staining (Figure 10D). Larger areas of dark blue staining (indicating more collagen deposition) were found in the other groups compared to P/SA/GO (NIR), indicating a higher rate of scar tissue formation [73]. This suggests that P/SA/GO (NIR) contributes to the normalization of skin regeneration, rather than the excessive proliferation of fibroblasts leading to scar formation. At the same time, rich angiogenesis is facilitating the entry of nutrients, immune cells and oxygen into the wound to accelerate wound healing [74]. So CD31 immunohistochemical staining was performed to assess the level of angiogenesis in the regenerated tissue (Figure 10E). Compared with the control group, there was a significant increase in CD31-positive cells in the hydrogel group. It indicates that the hydrogel group promoted angiogenesis and accelerated wound healing. These results confirmed that P/SA/GO (NIR) greatly promoted the seamless healing of wounds.

In summary, PNIPAM/SA/GO has adjustable contraction characteristics and controllable local tissue adhesion, and uses NIR stimulation to regulate the contraction of hydrogel to achieve control of shrinkage rate. Meanwhile, due to the biological function of SA, it can significantly promote wound closure and scar free healing. These results suggest that P/SA/GO provides a candidate material that can be used as a wound dressing to accelerate wound closure through a suture-free strategy and to promote wound traceless healing through mechanical stimulation. However, in practical applications, patient age and patient site also need to be considered. In addition, more animal models are needed to further test treatment effects.

## Conclusion

A double crosslinking network of hydrogels based on PNIPAM and SA was prepared by free radical polymerization and calcium ion crosslinking. By dispersing the GO component, excellent NIR properties were obtained, and temperature-sensitive self-shrinking hydrogels that could be adjusted by NIR were prepared successfully. In addition, through the introduction of a bridging polymer (CS), the hydrogel obtains regionally adjustable adhesion properties. After P/SA/GO (NIR) is used as a wound dressing to treat full-layer skin defects *in vivo*, it can accelerate wound scar healing by transmitting certain mechanical stimulation, blocking tension interference, inhibiting inflammatory response, and promoting angiogenesis and granulation tissue formation. It indicates that the developed flexible adhesive adjustable self-shrinking dressing can meet the current market demand for non-traceable dressing, and can provide a reference for future research on non-traceable dressing.

## Supplementary Material

rbad081_Supplementary_Data
