# Supplementary material for: Double-crosslinked PNIPAM-based hydrogel dressings with adjustable adhesion and contractility
Source: Regen Biomater. 2023 Sep 9;10:rbad081. doi: 10.1093/rb/rbad081 (PMC10570987; doi:10.1093/rb/rbad081)
Supplement: rbad081_Supplementary_Data [file rbad081_supplementary_data.zip › Supporting Information.docx]

Supporting Information:Figures S1-S6

**Double-crosslinked PNIPAM-based hydrogel dressings with**

**adjustable adhesion and contractility**

Yu Cao^a^, Longfei Wang^a,b*^, Xiumei Zhang^a^, Yi Lu^a^, Yan Wei^a,b^, Ziwei Liang^a,b^, Yinchun Hu^a,b^, Di Huang^a,b*^

a Research Center for Nano-Biomaterials & Regenerative Medicine, Department of Biomedical Engineering, College of Biomedical Engineering, Taiyuan University of Technology, Taiyuan 030024, China

b Shanxi-Zheda Institute of Advanced Materials and Chemical Engineering, Taiyuan 030032, China

*Corresponding to: [huangjw2067@163.com](mailto:huangjw2067@163.com) (D. Huang) or [wanglongfei@tyut.edu.cn](mailto:wanglongfei@tyut.edu.cn) (L. Wang)

Telephone: 86-351-3176650

**Keywords**: hydrogel dressings, temperature-sensitive, adjustable adhesion, adjustable contractility, scar-free healing


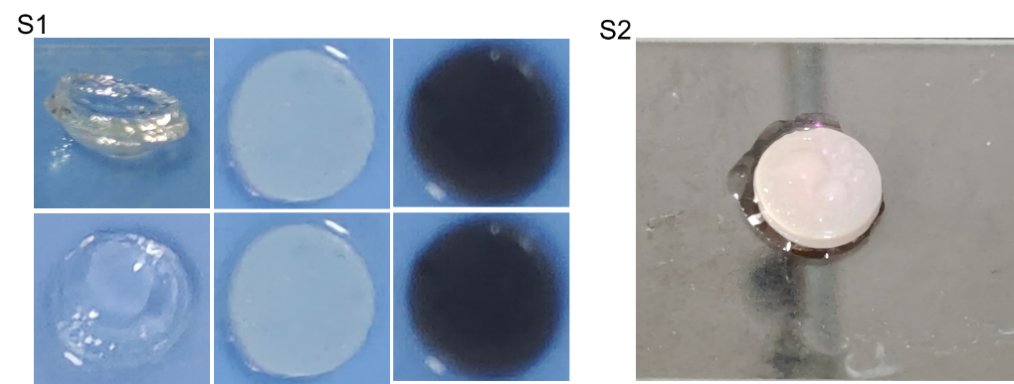


Fig S1. Physical drawing after three cycles at 37 °C and 25 °C.


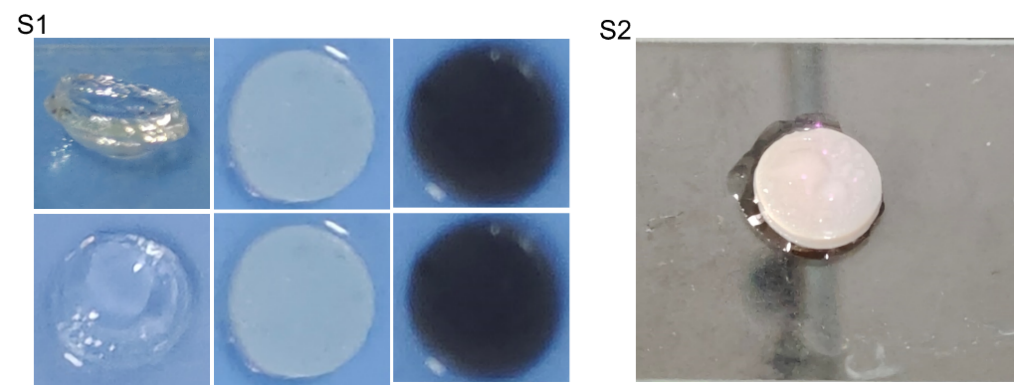


Fig S2. Whitening image of P/SA/FGO hydrogel under NIR irradiation.


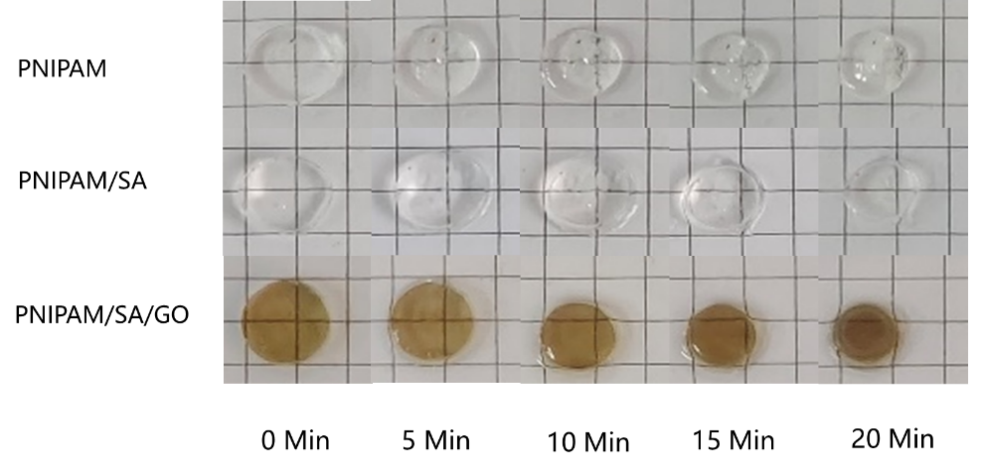


.

Fig S3. Physical images of hydrogels exposed to 1w NIR for different periods of time.


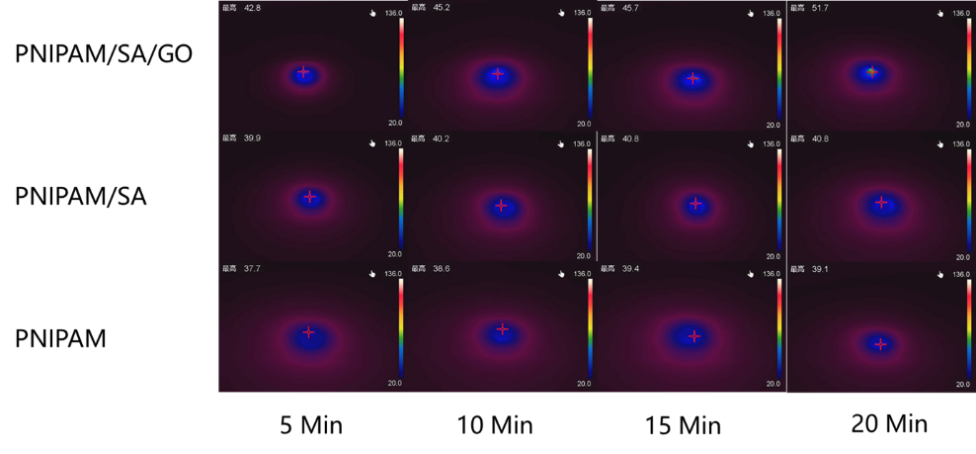


Fig S4. Infrared thermal imaging of hydrogels at different time periods under 1w NIR irradiation.


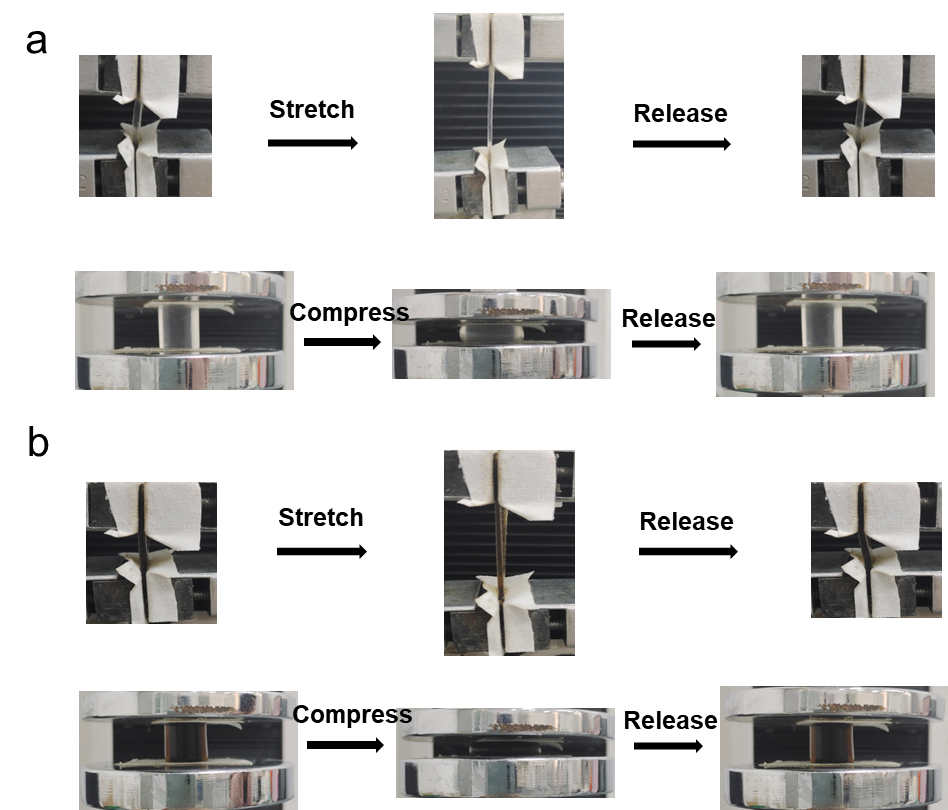


Fig S5. a. Physical illustration of a stretching and compression cycle of the P/SA hydrogel. b. Physical illustration of a stretching and compression cycle of the P/SA/GO hydrogel.


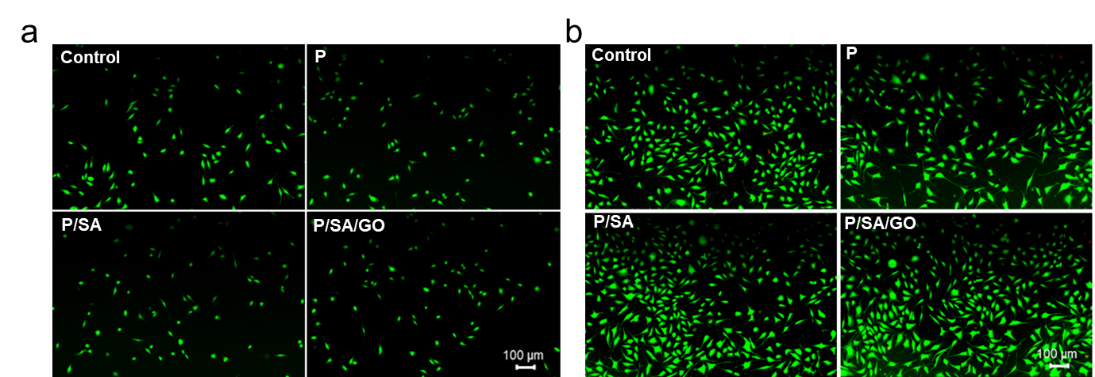


Fig S6. a. Live/dead fluorescent staining images of fibroblasts at 1 day. b. Live/dead fluorescent staining images of fibroblasts at 3 days.
